# Sleep Recovery Improves Total Sleep Deprivation‐Induced Cognitive Deficits and Histoarchitectural Distortions in the CA3 of Adult Wistar Rats Via Modulation of Oxidative Stress Pathway

**DOI:** 10.1002/alz70855_096926

**Published:** 2025-12-23

**Authors:** Ubong Udeme Ekpo, Uduak Emmanuel Umana, Adamu Abubakar Sadeeq, Sonhap James Sambo

**Affiliations:** ^1^ Ahmadu Bello University, Zaria, Zaria, Kaduna, Nigeria; ^2^ Ahmadu Bello University, Zaria, Zaria, Kaduna State, Nigeria

## Abstract

**Background:**

Sleep deprivation is a serious health concern that impairs daily functioning and well‐being. Prolonged lack of sleep significantly reduces cognitive function and productivity. This study investigated the restorative effects of sleep recovery after total sleep deprivation on cognitive function and the histoarchitecture of the CA3 region in adult male Wistar rats.

**Method:**

Twenty‐four adult male Wistar rats were divided into four groups of six. Group I served as the control and remained in their home cages, while Groups II, III, and IV underwent 24 hours of sleep deprivation daily for five days. After this, Groups III and IV had recovery periods of seven and twenty‐one days, respectively, in their home cages. Spatial memory was evaluated using the Morris water maze test. Rats from each group were humanely sacrificed at the end of the experiment. Their brains were harvested and divided into two halves. One half was homogenized to assess oxidative stress biomarkers, while the other was used for histological studies.

**Result:**

The sleep recovery groups exhibited a significant increase (*p* < 0.05) in latency time during the Morris water maze probe test, compared to the sleep deprivation‐only group. Oxidative stress analysis indicated a significant decrease (*p* < 0.05) in Malondialdehyde levels and increased superoxide dismutase, catalase, and glutathione concentrations in the sleep recovery groups. Additionally, sleep recovery groups showed lower expression of amyloid‐beta protein plagues and improved histological changes in the CA3 compared to the group that underwent only sleep deprivation.

**Conclusion:**

Sleep recovery reduced the accumulation of beta‐amyloid plaques, enhanced cognitive function, and improved histoarchitectural changes in the CA3 region by decreasing the production of reactive oxygen species (ROS) and improving the clearance of damaged cells

